# High prevalence of *Toxoplasma gondii* oocyst shedding in stray and pet cats (*Felis catus*) in Virginia, United States

**DOI:** 10.1186/1756-3305-6-266

**Published:** 2013-09-17

**Authors:** Emily L Lilly, Caroline D Wortham

**Affiliations:** 1 Biology Department, Virginia Military Institute, Lexington, VA 24450, USA

**Keywords:** Cat, Fecal, *Felis catus*, Molecular detection, Oocyst, Parasite, PCR, Prevalence, *Toxoplasma gondii*

## Abstract

**Background:**

The protozoan *Toxoplasma gondii* is the causative agent of toxoplasmosis, with complications varying from mental disease to death. While human infection can occur via ingestion of tissue cysts from infected meat, most human infection comes from oocysts. Cats are the only definitive host, and thus shedding of oocysts by cats provides the ultimate source of toxoplasmosis.

**Methods:**

While most studies in the area use seroprevalence to monitor *Toxoplasma* incidence in cat populations, this provides only a history of infection. This study used PCR detection of oocysts from cat feces to more accurately estimate the numbers of cats producing oocysts and thus posing an active health risk. DNA sequencing was use to confirm the identity of the PCR products.

**Results:**

Of the 49 cats tested, 9 yielded PCR products of the expected size. Six of the nine were determined by sequence analysis to be false positives, while three products were true positives. Overall, 6% of cats examined were found to be actively shedding oocysts.

**Conclusions:**

The incidence of oocyst shedding in the cat population studied was significantly higher than expected and higher than found in most cat populations world-wide. Of equal importance, the primers tested were shown to produce PCR products of multiple sizes and non-target products of expected size. We detected false positives at a higher rate than true positives, emphasizing the need for confirmatory analysis. Further research may produce better protocols for *Toxoplasma* detection from cat fecal samples.

## Background

Toxoplasmosis, caused by the protozoan *Toxoplasma gondii*, can lead to encephalitis, retinitis, and myocarditis [1,2]. Among immuno-compromised individuals, toxoplasmosis is a leading cause of hospitalization and death [3]. Congenital toxoplasmosis affects an estimated 5000 newborns each year in the United States [4], resulting in prematurity, mental retardation, and ocular disease [5]. Acute maternal infection can also result in fetal loss or neonate death [5,6]. This is particularly concerning given that 85-89% of childbearing age women have no immunity to *T. gondii*[7], and are thus at risk of developing an acute infection if exposed to the parasite.

Chronic infection occurs in approximately 22.5% of the US population [7]. The latent infection appears to pose no direct physical health risks, but it has been associated with increased rates of disturbing behavior, including homicide [8], suicide and other self-directed violence [9], as well as increased incidence of schizophrenia and other neuropsychiatric diseases [10].

Primary routes of acute human *T. gondii* infection include ingestion of tissue cysts in undercooked, contaminated meat, congenital infection through the placenta, and ingestion of oocysts from soil, water, or cat litter [7,11]. Oocysts are produced by *T. gondii* only through sexual reproduction in its definitive host, the cat [11-13]. Oocysts are shed in cat feces and can remain viable in soil and water samples for months to years [14]. Individuals with occupations requiring contact with soil in environments frequented by cats are significantly more likely to contract toxoplasmosis [7]. However, the more significant risk factor is contact with cats and cat litter. Owning just one cat increases the risk of toxoplasmosis, but having three or more kittens makes an individual over 70 times more likely to become infected with *T. gondii*[4]. While acute infection can be fatal to young kittens [12], cats may be asymptomatic [11], increasing the likelihood of accidental infection.

*Toxoplasma gondii* is ubiquitous in Virginia. Antibodies to *T. gondii* were present in the sera of 27% of tested lambs [15], 20% of tested dogs [16], and 27% of tested cats [17]. However, documentation of *T. gondii* antibodies could indicate prior exposure or a latent infection in which *T. gondii* is present only in tissue cysts [18]. In food animals such as lambs, tissue cysts represent a potential health risk for humans through meat consumption [15], but they are unlikely to pose a risk in a non-food animal like the cat. In cats, danger of infection exists only when the animal is actively shedding oocysts [11].

The majority of oocysts are produced shortly after the initial acquisition of the parasite, peaking within a month of initial infection [11,12]. Oocyte shedding generally lasts no more than 21 days [11,19], although it may recur with immunosuppression [20]. In comparative studies of blood serum and fecal assays, 0% [21-25], 3% [26], 4% [27], and 6% [28] of seropositive cats were found to have oocyte-contaminated feces. Thus, while serological testing may be applicable for determining parasite exposure, it is likely to vastly overestimate human health risk from cats.

Methods for determining oocyte presence in fecal samples include microscopy, mouse bioassay, and PCR [11,13]. Microscopy is time consuming and requires specialized training to visually identify *T. gondii* oocysts [13]. In addition, cyst-forming organisms with similar morphology must be differentiated with subsequent tests [11,19]. Mouse bioassay is more sensitive than microscopy [28], but requires the use of live mice and amplifies *T. gondii*, posing a biohazard. PCR is the most time-efficient of the three, requires only common molecular biology experience, easily differentiates *T. gondii* from other cyst forming eukaryotic parasites, and is highly sensitive [13,27].

In this study, two previously developed sets of PCR primers [13,29-31] were used in combination with sequence confirmation to determine oocyte presence in cat feces collected throughout Rockbridge County, VA.

## Methods

### Fecal samples

Sixty unique fecal samples were collected from both pet and stray cats in Rockbridge County, VA. Domestic environmental conditions of the pet cats were defined as “indoor only,” or “both indoor and outdoor.” Cats living outside only were categorized as “stray.”

### Coprologic diagnosis by PCR

DNA was extracted from each sample using the QIAamp® DNA Stool Mini Kit (Qiagen), with modifications to the extraction protocol as previously described [13]. Two sets of *T. gondii* specific primers (Table 1) were used in separate PCR reactions for every sample. Amplification followed the methodology of the reference paper. Universal bacterial 16S rRNA gene primers 27F and 1492R were used as positive controls to ensure that DNA extracts contained amplifiable DNA and were free of PCR inhibitors.

**Table 1:** **Primers used for PCR detection of *Toxoplasma gondii *oocysts in fecal samples**

**Primer pair**	**TOX4/TOX5**	**TOXF/TOXR**
Forward sequence	5’-CGCTGCAGGGAGGAAGACGAAAGTTG-3’	5’-GGAGGACTGGCAACCTGGTGTCG-3’
Reverse sequence	5’-CGCTGCAGACACAGTGCATCTGGATT-3’	5’-TTGTTTCACCCGGACCGTTTAGCAG-3’
Expected product size	529 bp	129 bp
Reference	[13,30,31]	[29]

Sequences of primers used in this study to detect *Toxoplasma gondii.*

### Post amplification analysis

PCR products were separated on agarose gels. When multiple bands were observed, bands of appropriate size for the primer set used were excised and extracted using QIAquick® Gel Extraction. The purified products were sent for commercial DNA sequencing. The chromatographs were analyzed by hand for accuracy and the resulting sequences were subjected to nucleotide BLAST analysis [32].

## Results

Positive control reactions amplified bacterial DNA from 49 of the fecal samples; 48% of which were from stray cats; 33% were from strictly indoor pets; and 11% from cats with both indoor and outdoor access. Gel electrophoresis showed that many of the PCR products produced with both primer sets, (up to 8–10 per sample), were not of the appropriate size, indicating non-specific amplification. Nine of the samples (18%) yielded bands of predicted size, which were excised and sequenced. There was a high incidence of false positives: 6 of the 9 bands of appropriate size produced sequences identified as common fecal bacteria. Three samples, (from 1 stray and 2 indoor/outdoor pets), yielded sequences with high identity to known *Toxoplasma gondii* isolates, and were identified as positive for *T. gondii* oocytes. Thus, the incidence of oocyte shedding was determined to be 6% (3/49). One of the positive samples yielded positive results with both primer sets; the remaining two produced *T. gondii* products only with the primers developed by Costa *et al.*[29].

### Comparative analysis

Twelve recent studies provided thirteen values for the percentage of cat fecal samples testing positive for *Toxoplasma gondii* oocysts (Table 2). For each study, a χ^2^ test was used to determine whether there were a statistically different number of positive fecal samples in Rockbridge County, VA, than would have been expected had the percent positive matched the reference study. The percent positive in this study (6%) was significantly higher than expected when compared with 10 of the samples, comparable with 2, and less than expected when compared with the feral cat population in Ethiopia (when assayed by mouse bioassay, Table 2).

**Table 2:** **Comparison of the current study (6% positive) with prior studies on *Toxoplasma gondii *oocysts in cat feces**

**Location**	**Cat type**	**Method**	**Fecal positives (%)**	**Difference from current**	**Significance (χ2 test)**	**Reference**
China	Stray	Mouse bioassay	0%	Less	p = 0	[22]
China	Stray	Mouse bioassay	0%	Less	p = 0	[20]
Egypt	Stray	Mouse bioassay	0%	Less	p = 0	[21]
Spain	Stray and pet	Microscopy	0%	Less	p = 0	[18]
Colombia	Stray	Mouse bioassay	0%	Less	p = 0	[19]
Europe	Stray and pet	Microscopy	0.11%	Less	p = 0	[33]
Switzerland	Stray and pet	PCR	0.30%	Less	p = 0	[34]
United States (CA)	Stray and pet	Microscopy	0.90%	Less	p = 0.0001	[35]
Canada (PEI)	Stray	Microscopy	1.30%	Less	p = 0.003	[27]
Finland	Stray	Microscopy/PCR	1.50%	Less	p = 0.008	[26]
Ethiopia^a^	Stray	Microscopy	5.50%	Comparable	p = 0.85	[28]
Italy	Stray	PCR	16%	Comparable	p = 0.06	[36]
Ethiopia^a^	Stray	Mouse bioassay	22.20%	More	p = 0.007	[28]

^a^The same cats were tested by both microscopy and mouse bioassay, with greater detection using mouse bioassay.

Comparison between current data and other measures of oocyst shedding in cat feces.

## Discussion

The prevalence of *Toxoplasma gondii* oocyst shedding among the cat population in Rockbridge County, VA, was significantly greater than 10 of the 12 reference populations (Table 2). The only sample statistically higher than the current study was a population of feral cats from Ethiopia, where, 16% of cats tested copropositive by PCR, 22% tested copropositive by mouse bioassay, and 92% of cats tested seropositive for *T. gondii* antibodies, which is far above the world average of 30-40% seropositive cats [11].

*T. gondii* in Rockbridge County warrants further study to confirm the high prevalence and to determine potential causes and human impacts. In addition to studying cats, it would be prudent to sample soil and water as well, given that environmental *T. gondii* concentrations are higher when infected cats are present [37,38].

Of equal concern, both primer sets published as specific to and diagnostic for *Toxoplasma gondii* yielded a substantial number of false positives. Fifty percent of PCR products of relevant size were shown to be derived from fecal bacteria upon full sequence analysis. Additionally, multiple bands of irrelevant size were also produced, although PCR parameters were as described in the reference studies [13,29,31]. The false positives emphasize the need to use a second confirmation method on every apparently positive product. Gel electrophoresis can quickly eliminate products of vastly different size than expected, but a second method is required. Sibley *et al.*, [39] used restriction fragment length polymorphism (RFLP) and nested-PCR to confirm *T. gondii* DNA sequence in PCR products. However, full sequencing provides a greater amount of data than RFLP, potentially yielding data for phylogenetic comparisons. Because of the ease and accuracy of DNA sequencing, it is recommended that similar studies use this method to confirm *T. gondii* presence and improve detection techniques.

## Conclusions

The incidence of oocyst shedding in the cat population studied was significantly higher than expected and higher than found in most cat populations world-wide. Further research should be conducted to determine if the high prevalence of oocyst shedding cats is typical of the area. If so, measures in public education of at-risk groups may be warranted. Of broad importance, the primers tested were shown to produce false positives at a higher rate than true positives, emphasizing the need for confirmatory DNA sequence analysis. Current primers are tested against other common pathogens for use in clinical detection, but not against the numerous bacteria typically present in cat feces. Further research may produce better protocols for *Toxoplasma* detection from cat fecal samples.

## Competing interests

Neither of the authors has any conflict of interests concerning the work reported in this paper.

## Authors’ contributions

CDW and ELL conceived and designed the experiment. CDW collected samples, performed molecular analyses, and helped to draft the manuscript. ELL conducted the sequence analysis and comparative prevalence analyses, and completed the manuscript. Both authors approved the final manuscript.
